# A case of undifferentiated-type mucosal gastric cancer with multiple lymph node metastases fulfilling the curative criteria for endoscopic resection according to routine pathological analysis

**DOI:** 10.1186/s40792-016-0225-7

**Published:** 2016-09-13

**Authors:** Takao Hara, Tsuyoshi Etoh, Yoshitake Ueda, Yuki Shitomi, Hidefumi Shiroshita, Norio Shiraishi, Tsutomu Daa, Masafumi Inomata

**Affiliations:** 1 Department of Gastroenterological and Pediatric Surgery, Oita University Faculty of Medicine, Hasama-machi, Oita, 879-5593 Japan; 2 Center for Community Medicine, Oita University Faculty of Medicine, Oita, Japan; 3 Department of Diagnostic Pathology, Oita University Faculty of Medicine, Oita, Japan

**Keywords:** Undifferentiated-type gastric cancer, Endoscopic submucosal dissection, Lymph node metastasis

## Abstract

**Background:**

Endoscopic resection is accepted as the standard treatment for early mucosal gastric cancer, and its indications have recently been expanded while its long-term outcomes are still unclear. Herein, we present a didactic case of undifferentiated-type mucosal gastric cancer fulfilling the expanded indication and curative criteria for endoscopic submucosal dissection (ESD), having synchronous multiple lymph node metastases.

**Case presentation:**

A 40-year-old woman was found to have a *Helicobacter pylori* infection at a standard health check with no abdominal symptoms. She received an upper gastrointestinal endoscopy and found to have an undifferentiated-type mucosal gastric cancer with the size of 15 mm in diameter without ulceration, which fulfilled the expanded indication for ESD. According to patient’s preference, we performed laparoscopy-assisted distal gastrectomy with D1+ lymph node dissection, and routine pathological analysis revealed a predominantly signet ring cell carcinoma limited to the mucosa without ulceration or any vessel involvement; on the other hand, 15 lymph node metastases were detected. Then, we added deep sectioning of the whole tumoral area at a thickness of 20 μm and immunohistochemical analyses. As the result, an isolated lymphatic capillary involvement of the extremely superficial submucosa was identified in a single histological section, and pathological diagnosis was corrected to ly1. She received postoperative adjuvant chemotherapy with an S-1 oral agent and had no recurrence under strict surveillance for 1 year postoperatively.

**Conclusions:**

When we perform ESD for undifferentiated-type gastric cancer, deep sectioning of the whole tumoral area into thin slices and immunohistochemical staining using D2-40 should be practically considered.

## Background

Endoscopic resection has been accepted as a standard treatment for early mucosal gastric cancer, associated with a very low incidence of lymph node metastasis [1]. According to recent Japanese gastric cancer treatment guidelines [2, 3], the presence of differentiated-type mucosal gastric cancer up to 20 mm in diameter without ulceration is an absolute indication for endoscopic submucosal dissection (ESD), while the presence of undifferentiated-type mucosal gastric cancer up to 20 mm in diameter without ulceration is specified as an expanded indication for ESD.

Although the expanded indication for ESD for the treatment of early gastric cancer is based on the assumption of negligible lymph node involvement, the incidence of lymph node metastasis in cases of early gastric cancer with expanded indication is higher than that in cases where there is an absolute indication for ESD [4]. In contrast, studies have reported a comparable low risk of lymph node metastasis in cases with an absolute indication for endoscopic resection, compared to cases with expanded indication [5]. Moreover, ESD for expanded indication is reported to be technically feasible from the aspect of short-term outcome [6, 7]. In Japan, a phase II clinical trial was recently conducted to evaluate the efficacy of ESD for intramucosal gastric cancer of undifferentiated type, with a primary end point of 5-year overall survival [8]. At present, the use of ESD in cases with expanded indication remains controversial in terms of long-term outcome. Here, we report a case of undifferentiated mucosal gastric cancer with multiple lymph node metastases fulfilling the expanded curative criteria for endoscopic resection according to routine pathological analysis.

## Case presentation

**Fig. 1:**
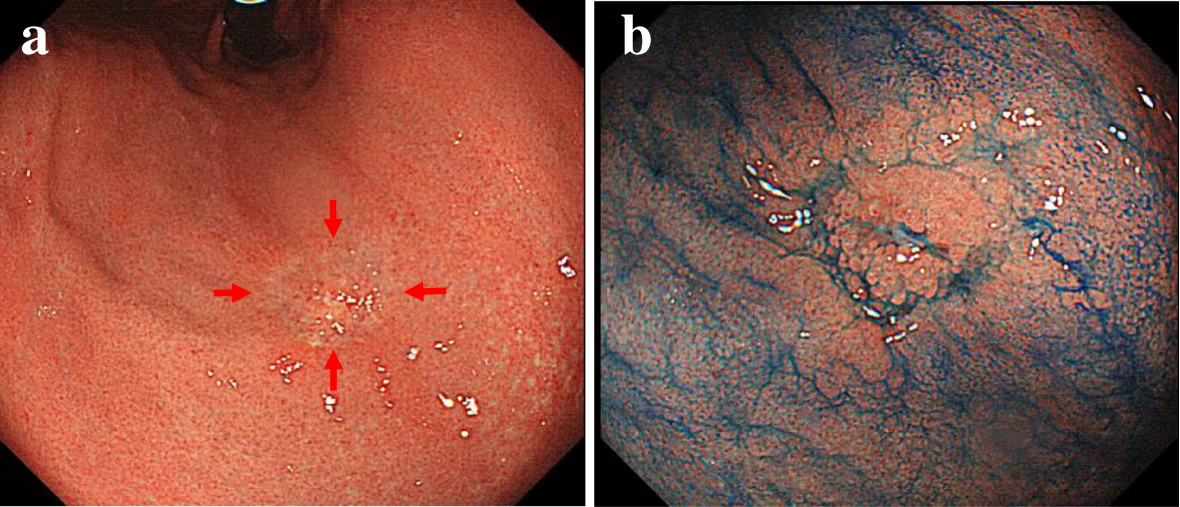
Upper gastrointestinal endoscopic findings. **a** The 0-IIc-type lesion with 15 mm in diameter was located on the lesser curvature of the middle gastric body without ulceration (*arrow*). **b** A biopsy scar was found in the center of the lesion by endoscopy with indigo carmine

**Fig. 2:**
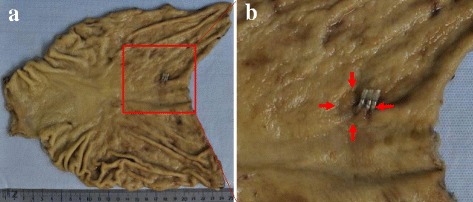
Macroscopic findings of the resected specimen. **a** A 0-IIc-type lesion of 15-mm diameter without ulceration was detected, and clips were applied to the oral side of the tumor. **b** Magnified view of the lesion (*arrow*)

A 40-year-old woman was found to have a *Helicobacter pylori* infection at a standard health check and was accordingly referred to our hospital. An upper gastrointestinal endoscopy demonstrated a 0-IIc-type lesion 15 mm in diameter located at the lesser curvature of the middle gastric body, without ulceration (Fig. 1). Histological analysis of a biopsy specimen demonstrated a signet ring cell carcinoma, while computed tomography revealed neither enlarged lymph nodes nor distant metastases. Accordingly, undifferentiated-type mucosal gastric cancer was diagnosed, with the possibility of lymph node metastasis considered very low. As the lesion fulfilled the expanded indication for ESD according to the Japanese gastric cancer treatment guidelines [3], we elected to perform ESD as recommended treatment. However, because the patient and her family ultimately requested surgical treatment, we therefore performed laparoscopy-assisted distal gastrectomy with D1+ lymph node dissection without any intraoperative complications. Macroscopically, the 0-IIc lesion without lymph node metastasis was identified in the resected specimen (Fig. 2).

Routine histological analysis of the resected specimen with hematoxylin and eosin (H&E) staining revealed a predominantly signet ring cell carcinoma of 15-mm diameter limited to the mucosa, without lymphatic-vascular capillary involvement or ulcerative components. There were neither apparent findings outside the zone of the tumoral area nor another malignant lesion in the resected specimen. Multiple lymph node metastases of the perigastric area were unexpectedly identified in 15 of the 45 retrieved lymph nodes; therefore, the tumor was classified as pathological stage IIB (T1a N3 M0).

**Fig. 3:**
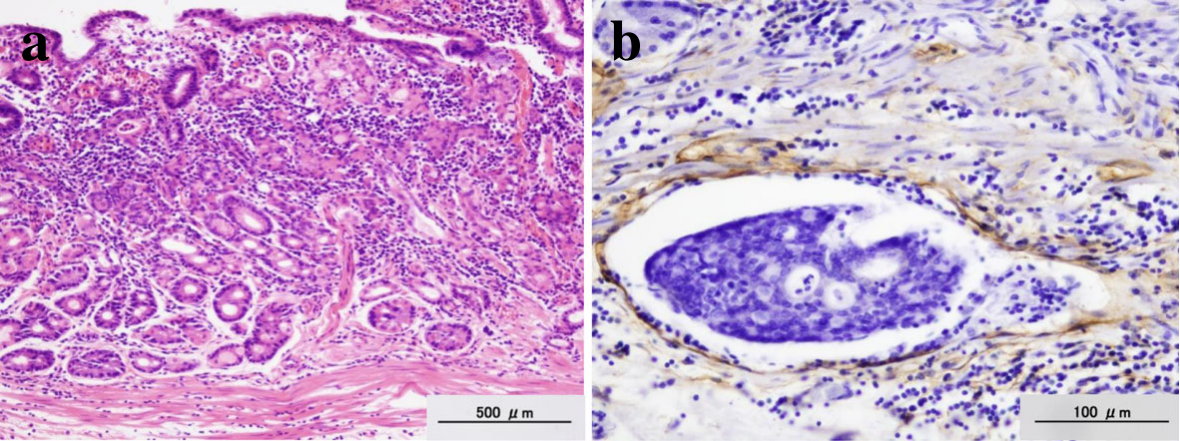
Microscopic findings of the resected specimen. **a** H&E staining revealed all cancer cells were completely limited to the mucosa without lymphatic-vascular capillary involvement or ulcerative components. **b** Immunohistochemical staining with D2-40 revealed isolated lymphatic capillary involvement of the extreme superficial submucosa in just one histological section

Because of multiple lymph node metastases, the present case was sufficiently unusual to prompt us to perform additional deep sectioning of the whole tumoral area at a thickness of 20 μm, and further analyses were performed using H&E and D2-40 staining. The results of these analyses demonstrated that all cancer cells existed completely limited to the mucosa, with immunohistochemical staining for desmin and vimentin revealing no evidence of fibrosis in the submucosal layer or deformity of the muscularis mucosae. However, we finally identified isolated lymphatic capillary involvement of the extremely superficial submucosa in a single histological section (Fig. 3).

The postoperative course of this case was uneventful, and the patient received postoperative adjuvant chemotherapy with an S-1 oral agent. At this moment, no recurrence was observed following strict surveillance for 1 year postoperatively.

In general, further size and ulceration of early mucosal gastric cancer are considered as risk factors for lymph node metastasis. The rate of lymph node metastasis from mucosal early gastric cancer has been reported to be as high as 8 %, and the risk factors for lymph node metastasis were tumor size and having ulceration in 148 cases, who were diagnosed with early mucosal gastric cancer without adaptation of endoscopic resection and underwent laparoscopic gastrectomy [4]. Hirasawa et al. reported that tumor sizes greater than 21 mm, lymphatic-vascular capillary involvement, and submucosal penetration were considered as risk factors for lymph node metastasis in a series of 3843 cases diagnosed with undifferentiated-type early gastric cancer who underwent gastrectomy [9]. Recently, Pyo et al. demonstrated tumor sizes larger than 17 mm, elevated tumor type, and lymphatic-vascular involvement to be significantly associated with lymph node metastasis [10]. The present case would be a candidate for curative resection with expanded criteria according to the Japanese guidelines because of the identification of an undifferentiated lesion with a diameter of 15 mm without ulceration. However, postoperative histological analysis revealed as many as 15 regional lymph node metastases in our case, despite the absence of metastatic lymph nodes in either preoperative analysis or intraoperative observations.

Synchronous lymph node metastasis has previously been reported in a case fulfilling the expanded criteria for endoscopic resection [11]. In this report, the lesion was sliced into 60 thin sections, and additional histological analysis was performed using D2-40, desmin, Masson, and vimentin immunohistochemistry. As the result, lymphatic vessel involvement was identified in the deep mucosal layer, and accordingly, the authors suggested that routine pathological analyses for undifferentiated-type gastric cancer are inadequate for determining curative resection. Sako et al. reported an improvement in the diagnostic accuracy of lymphatic vessel involvement by using an immunohistochemical method with D2-40 in the pathological analysis of early gastric cancer [12]. Furthermore, a correlation between lymph node micrometastasis and lymphatic invasion has previously been reported, with micrometastasis found to be more closely correlated with D2-40 than H&E staining [13, 14]. There are some immunohistochemical examinations for detecting lymphatic-vascular involvement. Actually, D2-40 staining is the most powerful tool to detect lymphatic-vascular involvement. As for other methods, lymphatic vessel endothelial hyaluronan receptor-1 (LYVE-1) and platelet and endothelial cell adhesion molecule 1 (PECAM1) are also available, but these are less common. Therefore, we conducted additional pathological analyses of deep-cut sections of the whole tumoral area at 20-μm thickness using H&E and D2-40 staining to identify cancer cells in unexpected areas. Despite tumoral depth in the remaining mucosa, we finally identified isolated lymphatic capillary involvement in the shallow submucosal layer in one histological section. Consequently, gastrectomy with lymph node dissection could contribute to a favorable outcome in this case.

## Conclusions

The findings of this study indicate that intensive pathological analyses using H&E and D2-40 staining in thin-cut histological sections should be considered in cases of undifferentiated-type gastric cancer, fulfilling the expanded indication for endoscopic resection in order to detect minute lymphatic capillary involvement.

## Consent

Written informed consent was obtained from the patient for publication of this case report and any accompanying images. A copy of the written consent is available for review by the Editor-in-Chief of this journal.

## Abbreviations

ESD: Endoscopic submucosal dissection
H&E: Hematoxylin and eosin
